# Sonar sound groups and increased terminal buzz duration reflect task complexity in hunting bats

**DOI:** 10.1038/srep21500

**Published:** 2016-02-09

**Authors:** Katrine Hulgard, John M. Ratcliffe

**Affiliations:** 1 Department of Biology, University of Southern Denmark, 5230 Odense, Denmark; 2 Department of Biology, University of Toronto Mississauga, ON L5L 1C6, Canada

## Abstract

More difficult tasks are generally regarded as such because they demand greater attention. Echolocators provide rare insight into this relationship because biosonar signals can be monitored. Here we show that bats produce longer terminal buzzes and more sonar sound groups during their approach to prey under presumably more difficult conditions. Specifically, we found Daubenton’s bats, *Myotis daubentonii*, produced longer buzzes when aerial-hawking versus water-trawling prey, but that bats taking revolving air- and water-borne prey produced more sonar sound groups than did the bats when taking stationary prey. Buzz duration and sonar sound groups have been suggested to be independent means by which bats attend to would-be targets and other objects of interest. We suggest that for attacking bats both should be considered as indicators of task difficulty and that the buzz is, essentially, an extended sonar sound group.

For humans and other animals, demands on sensorimotor integration and task complexity are generally positively related^1^,^2^,^3^,^4^. However, quantifying this relationship, particularly with respect to attention, is made difficult because available environmental information and sensory system activity are not easily monitored in freely moving individuals^1^,^2^,^4^. This may be especially true during predator-prey interactions, when the time from the onset of predator attack to prey capture, or escape, is often very short^2^,^5^. Active spatial sensory systems (e.g., echolocation) provide a window into these processes because the predator generates the information used for prey detection and localization and these signals, concurrent with the movements of predator and prey, can be recorded^5^. For echolocating bats, changes in call design are reliable indicators of attention to objects at different locations^6^. We suggest that greater call emission rates reflect greater demands on attention: the more echolocation calls produced per unit time, the more returning echoes per unit time there will be to process.

Laryngeal echolocating bats increase their echolocation call emission rate after they detect, and begin to close in on, air- and water-borne prey^5^,^6^. Griffin and colleagues^7^ described three phases of aerial hawking bat’s pursuit: the search, approach, and terminal buzz phases. In vespertilionoid bats the terminal phase has since been further divided into the ‘buzz I’ and ‘buzz II’ subphases^8^,^9^. At the onset of buzz I, the bat is thought to take a breath, then begin to produce calls at rates >90 calls/s. In buzz II, bats drop their echolocation call peak frequency by roughly an octave, relative to the PF of the search phase calls, and produce calls at rates >160 calls/s^9^. This combination of lower call PF and extremely fast call emission rate broadens the bat’s sonar beam and maximizes auditory scene updates, respectively. Both mechanisms should help to reduce the odds of prey escape^9^,^10^,^11^.

In bats the duration of the terminal buzz varies considerably, between species and within individuals^12^,^13^. For example, in the laboratory the duration of the buzz for the big brown bat, *Eptesicus fuscus*, decreases by a factor three (~300 ms to ~100 ms) when hunting suspended, stationary prey near clutter versus hunting in more open space^12^. In the field and lab, its buzz tends to be 500 ms in duration, as for most vespertilionid bats^5^,^9^,^12^. There are exceptions, however. Red bats, *Lasiurus borealis*, have been recorded producing buzzes >2 s long when pursuing insects outdoors^13^.

*Myotis daubentonii* is an aerial hawking and trawling bat, catching insects from and just over water surfaces^8^ and commonly observed hawking prey >1 m above water surfaces and in open areas^14^. We tested the hypothesis that the duration of the buzz would change according to the strategy (hawking versus trawling) and degree of difficulty of the task (moving versus stationary target).

We predicted that *M. daubentonii* would produce shorter buzzes when trawling water-borne prey because the bat is essentially solving a 2D problem, with no vertical element, as opposed to aerial hawking prey, which we deem a 3D problem. We make this assumption based on two premises: first, Siemers and colleagues^15^,^16^ proposed that prey detection should be easier when foraging for insects on the surface of smooth water, relative to prey detection in open air. Second, capture should be simplified as water-borne insects movements are constrained to a horizontal plane, while those of flying insects typically are not. We also predicted bats would produce longer buzzes when prey was moving as opposed to stationary, based on the assumption that intercepting a moving target is more difficult than capturing a stationary one, all else being equal. In light of our results, we discuss terminal buzz duration as a proxy for an attack’s level of difficulty and, in the context of past research, the role of echolocation call sonar sound groups (SSGs) for foraging bats.

## Results

**Figure 1:**
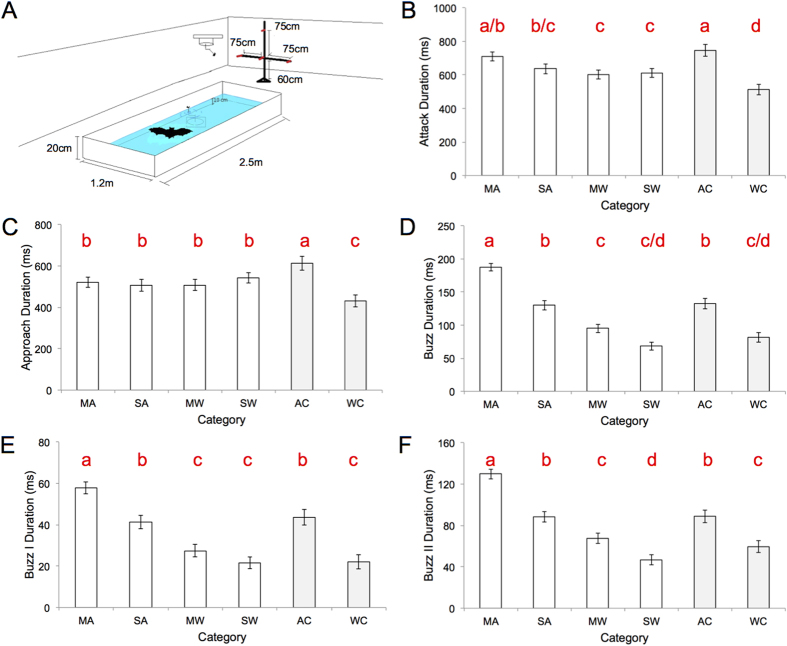
Flight room schematic and average (±SD) attack phase durations for all conditions. (**A**) Flight room arrangement: pond, tether, rotators, microphone array, (**B**) total attack duration, (**C**) approach duration, (**D**) total buzz duration, (**E**) buzz I duration, (**F**) buzz II duration. MA = moving air, SA = still air, MW = moving water, SW = still water, AC = air control, WC = water control. Bars that do not share the same red lower case letter are significantly different (Tukey HSD post-hoc comparisons).

**Figure 2:**
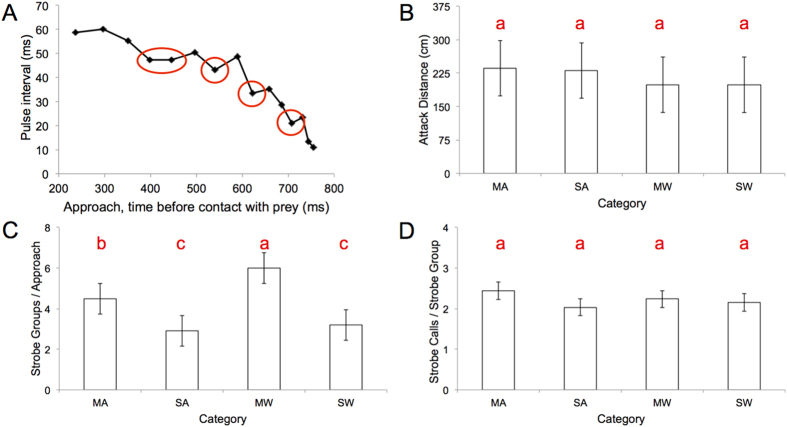
Call (pulse) intervals, attack distances, SSGs, spectrograms and oscillograms for attack sequences. (**A**) Approach phase call intervals, SSGs circled, for aerial attack on still prey, (**B**) average (±SD) distances covered during approach and buzz for experimental conditions, (**C**) average (±SD) number of SSGs produced during approach phase, (**D**) average (±SD) number of calls produced per SSG. Bars that do not share the same red lower case letter are significantly different (Tukey HSD post-hoc comparisons).

**Figure 3:**
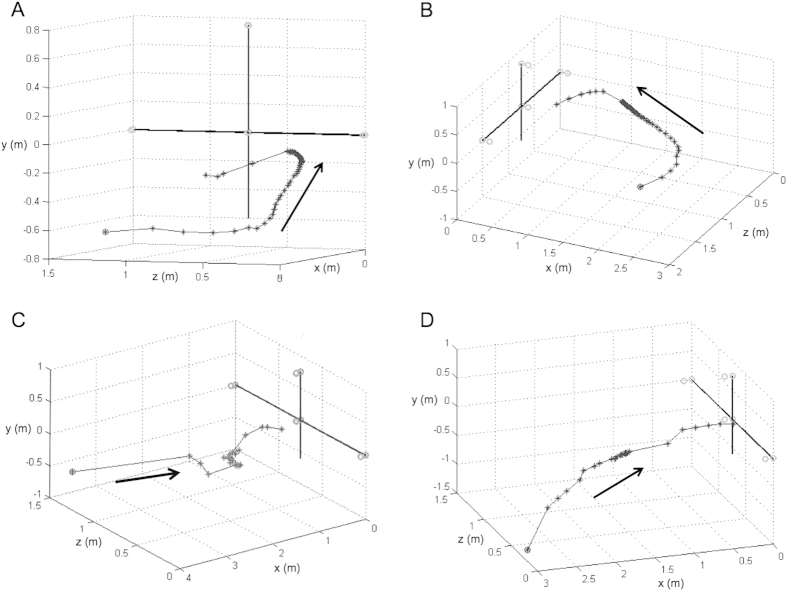
Representative 3-dimensional flight paths. (**A**) Moving air, (**B**) still air, (**C**) moving water, (**D**) still water.

We found that the time elapsed over the course of the entire attack sequence (i.e. estimated time of prey detection to time of capture) was significantly longer for the moving, airborne target category than the two experimental water categories, which did not differ from one another in this respect (ANOVA: *F*_5,16_ = 28.2, *P* < 0.0001, Tukey HSD post-hoc comparisons; Fig. 1B). This despite that fact that the distance from target at which the bat commenced its attack did not differ between experimental categories (ANOVA: *F*_3,36_ = 0.43, *P* = 0.73; Fig. 2B). We compared this direct line distance to the estimated distance travelled (measured using call-to-call emission positions) and found that the more circuitous flight paths taken by bats when tracking and intercepting moving airborne prey compared to stationary or water-borne prey accounted for this discrepancy (Fig. 3). That is, the difference between these two measures was significantly greater for attacks on moving airborne prey than that for the other experimental conditions which did not differ from one another (ANOVA: *F*_3,36_ = 9.3, *P* < 0.001, Tukey HSD post-hoc comparisons).

The duration of the approach phase did not differ between experimental categories (ANOVA: *F*_5,16_ = 13.6, *P* < 0.0001, Tukey HSD post-hoc comparisons; Fig. 1C); the number of SSGs produced during this phase did (ANOVA: *F*_3,36_ = 14.14, *P* < 0.0001, Tukey HSD post-hoc comparisons; Fig. 2C). Bats taking moving prey from water produced the greatest number of SSGs, followed by bats taking moving airborne prey. For hawking and trawling trials, bats produced more SSGs when prey was moving (Fig. 2C). Strobe calls per SSG did not differ between categories (ANOVA: *F*_3,36_ = 2.5, *P* = 0.08; Fig. 2D). Time elapsed from the first call of the first SSG until the last call of buzz II was greater when hawking moving prey than when trawling still prey (ANOVA: *F*_3,36_ = 3.6, *P* < 0.03; Tukey HSD post-hoc comparisons); the distance from this point to point of capture did not differ between categories (ANOVA: *F*_3,36_ = 0.8, *P* = 0.4).

Total buzz duration, and buzz I and buzz II sub-phases, was longest for bats when tracking moving, airborne prey; in turn, bats taking still, airborne prey produced longer buzzes, and buzz I and II sub-phases, than bats when trawling. Bats taking moving prey from water produced longer buzzes, and longer buzz II sub-phases, than bats taking still prey from water (Total buzz: ANOVA: *F*_5,16_ = 166.4, *P* < 0.0001, Tukey HSD post-hoc comparisons; Buzz I: ANOVA: *F*_5,16_ = 90.0, *P* < 0.0001, Tukey HSD post-hoc comparisons; Buzz II: ANOVA: *F*_5,16_ = 113.9, *P* < 0.0001, Tukey HSD post-hoc comparisons; Fig. 1). Detection distance and the duration of attack (and phases and sub-phases therein) are likely related; however, if sequential Bonferonni correction were to be applied^17^, all significant ANOVA test results (α = 0.05) would remain significant.

## Discussion

Buzz duration (and buzz I and buzz II sub-phases) varied considerably (~30 ms–~400 ms) and was related to differences in foraging task (Figs 1 and 2). We consider trawling as bats hunting in 2-dimensions, the water surface a plane on which insects are reliably found. Hawking, conversely, likely poses a bigger challenge. Here prey position can vary in 3-dimensions. Indeed we saw less direct flight trajectories when bats took moving prey in mid-air than when trawling and, that when doing so, they produced significantly longer buzzes (Fig. 3). Our prediction that the duration of the buzz would be greater for moving versus still prey was also confirmed (Fig. 1). Overall we found support for our hypothesis that greater task complexity would equate to longer buzz durations. We suggest buzz duration might be a reliable proxy for degree of difficulty of a given foraging task. Based on our results, we also speculate that during Buzz I the bat attempts to “lock on” to its target, a new hypothesis supported by longer Buzz I sub-phases when bats are tracking moving airborne prey overall, and the longer Buzz I sub-phases observed for bats hawking still prey as opposed to trawling. We recognize that longer Buzz II durations for moving prey may, in some of our trials, be partially due to the prey moving away from the bat after the buzz phase has been initiated.

Until recently, there has been some disagreement as to whether bats use the information in buzz echoes in real-time, or as data on capture success or failure for future assessment^18^. Recent results show that Daubenton’s bats are able to use information contained in buzz echoes in real-time and make sensorimotor adjustments for capture in <20 ms^18^. *Myotis lucifugus* produces a buzz before a landing, again indicating its importance in motor control^19^. *E. fuscus* produces its shortest buzzes when taking still, tethered prey within clutter and its prey capture success rate decreases as target-clutter distances decrease^12^. Similarly, *M. nattereri* uses very short buzzes when taking mealworms suspended close beneath baseball-sized objects^20^. Buzzing near clutter risks call-echo and target echo-clutter echo overlap^6^,^12^, perhaps explaining this otherwise apparently maladaptive reduction in information up-date.

In our study, vertical clutter was low for, and similar across, all conditions and level of difficulty varied only with respect to whether the prey was still or moving, on the water surface, or in mid-air. The similar attack distances and durations across conditions suggest that the durations of the buzz and approach phases are traded-off against one another, and buzzes are likely the more expensive to produce^9^,^11^. Buzz II calls are stable in interval (~6 ms) and these intervals are shorter than those before and after them, consistent with an SSG^6^,^12^,^20^,^21^. We therefore propose that the terminal buzz is essentially a long, call-rich SSG, with a broad sonar beam.

For *E. fuscus* aerial hawking stationary, suspended mealworms in the laboratory, bats use shorter buzzes but produce SSGs more often the greater the level of clutter^12^. Our results show that in comparatively open space, bats produce longer buzzes the more complex the task (hawking versus trawling, moving versus still prey), but use most SSGs when trawling moving prey. This suggests that, as *E. fuscus* does to clutter when hawking near to it^12^, trawling Daubenton’s bats may be attending to ripples on the water surface as they home in on prey. As for total attack duration, the time elapsed from the first call of the first SSG to the last call of the buzz was longer for hawking attacks on moving prey than trawling attacks on still prey; distance at this point did not differ between groups. This suggests that the first SSG, produced midway through the approach phase, might indicate the point when the bat changes from a strategy of approaching the prey, to plotting a course for its interception^22^.

While the purpose of our study was not to test the hypothesis that for trawling bats still water acts as an acoustic mirror - extending the echolocator’s prey detection distance - our results do not support it. Siemers and colleagues^15^ proposed the acoustic mirror hypothesis based on behavioural and ensonification experiments, providing further support through additional behavioural experiments^16^. However, in these studies water was not used. Instead linoleum was and we suggest that linoleum may not be a valid substitute for water. For one, mealworms are suspended in water, but sit atop linoleum. For two, while the water surface of our laboratory-based pond was as still as would ever be found in nature, this surface was never as “still”, and therefore reflective, as linoleum sheeting would be.

Our studies also differed in how we defined the point at which the bat detected the mealworm and this too could account for our different results. We used the original definition of Griffin and colleagues^7^, based on experiments using the congeneric, *Myotis lucifugus*, and modified for *M. daubentonii* (as described in [8]). Siemers and colleagues^16^ took a different approach, using a marked drop in call duration as an indicator of prey detection. Neither means is without its faults. In closing, we reiterate that the purpose of our experiments was not to test the water as acoustic mirror hypothesis. However, our results do indicate that it should be tested again, using prey suspended on the surface of water (and just above water) and new and improved means of estimating when a bat has detected prey.

## Materials and Methods

### Animals and ethics statement

We used 6 wild-caught, adult Daubenton’s bats (*Myotis duabentonii*). Experiments were conducted at the University of Southern Denmark in an indoor, screened flight room (L7 m*W4.8 m*H2.5 m, Fig. 1A). Centred in the room was a 10 cm deep pool of water (L2.5 m*W1.2 m; Fig. 1A). When not being flown, bats were housed in aluminium mesh cylinders (dia: 25 cm, h: 35 cm) with cloth hung from the top for roosting. The bats had continuous access to water, and ate >10 mealworms/day throughout their time in captivity. All experiments were conducted under a dim red light bulb (25 W). After experiments, bats were released at site of capture. Animal capture and experimentation was approved by Skov- og Naturstyrelsen and adhered to the legal requirements of Denmark and the institutional guidelines of the University of Southern Denmark.

### Experimental design

Two custom DC powered machines were used to move prey. One was positioned beneath the water surface and could rotate the floating, tethered mealworm on the water surface (radius: 20 cm, 18 rpm). The other was mounted on the ceiling and could rotate the tethered, hanging mealworm roughly 1.5 m from the floor (radius: 40 cm, 36 rpm). Floating and tethered mealworms were always both in the middle of the pond’s horizontal plane (Fig. 1A).

The bats were recorded hunting for prey in 4 experimental scenarios: (i) moving prey in mid-air (MA), (ii) still prey in mid-air (SA), (iii) moving prey on water (MW), (iv) still prey on water (SW). One prey item (a single mealworm, *Tenebrio molitor*) was available during each trial. The machine that rotated the prey in the MA category was not silent and so we had an air control category, where the prey was still, the machine moving (AC). We also conducted control trials for the water categories (WC), where the prey was still on the water surface and away from the machine, which rotated with a small piece of rubber submerged just beneath the water surface. This created a small ripple on the surface, like a moving mealworm. The sequence of experimental and control categories was randomized between bats. Aborted capture attempts were not analysed.

### Acoustic recording and analyses

We recorded the bats echolocation calls using an inverted T-shaped array of 4, ¼” G.R.A.S. microphones (G. R. A. S. Sound and Vibration, Holte, Denmark) placed at one of the far ends of the flight-room (Fig. 1A). The 3 horizontal microphones were 75 cm apart and 60 cm from the ground; the uppermost microphone was 75 cm above the centre microphone (Fig. 1A). The signals were amplified using Avisoft power modules (Avisoft, Berlin, Germany), digitized (Avisoft USGH1216 @ 300 kHz), and stored on a laptop computer. Files were 4 s and captured using a manual trigger when we observed the bat take the prey (2 s pre- and post-trigger). Microphones were calibrated using a G.R.A.S. 42 AB sound calibrator.

A total of 442 sound (.wav) files were analysed in BatSound (Petterssen Elektronik, Uppsala, Sweden). Pulse interval (pulse onset to onset of next pulse) was measured from oscillograms. The start of the approach phase (post-detection, onset of attack) was estimated as the first call with a pulse interval of ≤60 ms, the start of the buzz as first call with a pulse interval of ≤10 ms. Sonar sound groups (SSGs) were identified, and number of calls per SSG determined, as described in [21]. We had repeated measurements from individual bats, and so we included bat identity as a random effect to control for the effects of pseudoreplication.

Flight trajectories and the distance of the bat from the prey at the onset of attack were determined for a subset of recordings (*N* = 10 for each of the 4 experimental categories). Each call’s 3D position in space at time of emission was determined based on time of arrival differences at the 4 microphones in the array as described in [10]. We also determined the time and distance at which the first SSG was produced. All statistical analyses were conducted using JMP v12 (SAS Institute, Cary, North Carolina, USA).

## Additional Information

**How to cite this article**: Hulgard, K. and Ratcliffe, J. M. Sonar sound groups and increased terminal buzz duration reflect task complexity in hunting bats. *Sci. Rep.* **6**, 21500; doi: 10.1038/srep21500 (2016).
